# Evaluation of the Therapeutic Potential of Bone Marrow-Derived Myeloid Suppressor Cell (MDSC) Adoptive Transfer in Mouse Models of Autoimmunity and Allograft Rejection

**DOI:** 10.1371/journal.pone.0100013

**Published:** 2014-06-13

**Authors:** Lucile Drujont, Laura Carretero-Iglesia, Laurence Bouchet-Delbos, Gaelle Beriou, Emmanuel Merieau, Marcelo Hill, Yves Delneste, Maria Cristina Cuturi, Cedric Louvet

**Affiliations:** 1 ITUN, Inserm UMR_S 1064, Center for Research in Transplantation and Immunology, Nantes, France; 2 UMR Inserm 892 CNRS 6299, Université d’Angers, CHU Angers, Laboratoire d’Immunologie et Allergologie, Angers, France; Saint Louis University School of Medicine, United States of America

## Abstract

Therapeutic use of immunoregulatory cells represents a promising approach for the treatment of uncontrolled immunity. During the last decade, myeloid-derived suppressor cells (MDSC) have emerged as novel key regulatory players in the context of tumor growth, inflammation, transplantation or autoimmunity. Recently, MDSC have been successfully generated in vitro from naive mouse bone marrow cells or healthy human PBMCs using minimal cytokine combinations. In this study, we aimed to evaluate the potential of adoptive transfer of such cells to control auto- and allo-immunity in the mouse. Culture of bone marrow cells with GM-CSF and IL-6 consistently yielded a majority of CD11b^+^Gr1^hi/lo^ cells exhibiting strong inhibition of CD8^+^ T cell proliferation in vitro. However, adoptive transfer of these cells failed to alter antigen-specific CD8^+^ T cell proliferation and cytotoxicity in vivo. Furthermore, MDSC could not prevent the development of autoimmunity in a stringent model of type 1 diabetes. Rather, loading the cells prior to injection with a pancreatic neo-antigen peptide accelerated the development of the disease. Contrastingly, in a model of skin transplantation, repeated injection of MDSC or single injection of LPS-activated MDSC resulted in a significant prolongation of allograft survival. The beneficial effect of MDSC infusions on skin graft survival was paradoxically not explained by a decrease of donor-specific T cell response but associated with a systemic over-activation of T cells and antigen presenting cells, prominently in the spleen. Taken together, our results indicate that in vitro generated MDSC bear therapeutic potential but will require additional in vitro factors or adjunct immunosuppressive treatments to achieve safe and more robust immunomodulation upon adoptive transfer.

## Introduction

Myeloid-derived suppressor cells (MDSC) comprise a heterogeneous population of myeloid cells at various stages of differentiation accumulating during pathological situations, such as tumor development or inflammation, and with the ability to suppress T-cell responses [1], [2], [3]. In mice, MDSC are broadly defined as CD11b^+^ Gr1^+^ cells and have been shown to exhibit a variety of suppressor mechanisms [4], [5].

Growing evidence indicate a central role of MDSC in diverse models of autoimmune diseases [6] including type 1 diabetes [7], [8], arthritis [9], colitis [10], alopecia areata [11], myocarditis [12] or experimental autoimmune encephalomyelitis (EAE) [13], [14], [15]. A protective role of MDSC has also been documented in the context of allogenic transplantation [4], [16], [17], [18], [19], [20], [21]. Interestingly, a recent report linked the accumulation of MDSC with FoxP3^+^ regulatory T cells (Tregs) in kidney-transplanted patients [22].

Thus, similarly to Tregs [23], MDSC represent a novel regulatory cell type that could be manipulated to achieve immune tolerance in the context of autoimmunity or transplantation. Although injections of G-CSF [24], LPS [18] or IL-33 [25] have been shown to favor the generation of endogeneous MDSC in allograft recipient mice, a promising and clinically applicable approach would consist in the adoptive transfer of in vitro-generated MDSC. In this regard, the study by Rossner et al. initially paved the way towards MDSC generation from bone marrow (BM) cells using GM-CSF [26]. Alternatively, Zhou et al. demonstrated the development of MDSC from mouse stem cells [27]. Other studies reported that BM cells co-cultured with hepatic stellate cells could lead to the production of MDSC effectively preventing murine islet allograft rejection [28] or colitis [29]. Generally, GM-CSF, in conjunction with tumor cells conditioned culture medium, appeared as a pivotal cytokine for the generation of MDSC [30], [31]. IL-6 has subsequently been identified as a potent complement to GM-CSF for the generation of both mouse and human MDSC [32], [33]. Importantly, Marigo et al. showed that mouse bone marrow-derived MDSC generated with GM-CSF and IL-6 exhibit a stronger immunosuppressive activity in vivo and could induce long-term survival of pancreatic islet allograft upon repeated adoptive transfer [32]. This latter study opened an avenue to the generation of these cells in great numbers and in a controlled manner for their use in cellular immunotherapy.

In the current study, we investigated and compared the suppressive potential of BM-derived MDSC generated in vitro with GM-CSF and IL-6, without combination treatment, in different mouse models of auto- and allo-immunity.

## Results

**Figure 1:**
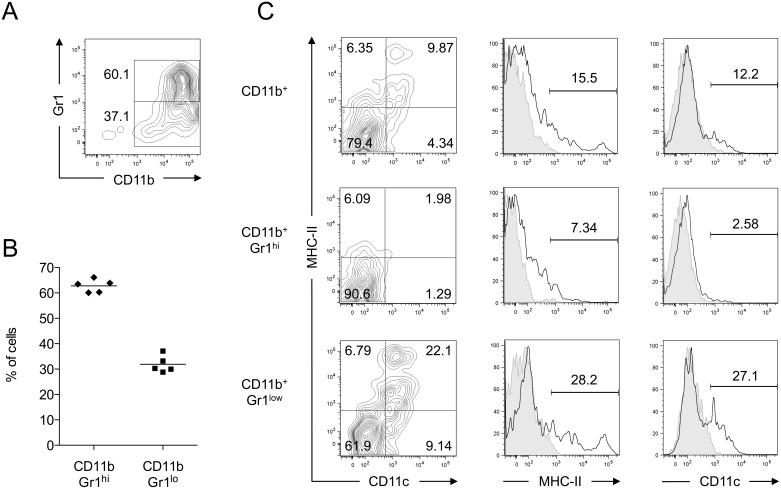
Phenotype of BM-derived MDSC. (A) BM cells from naive mice were cultured in the presence of GM-CSF and IL-6 for 4 days. Surface expression of CD11b and Gr1 was measured by flow cytometry. (B) Quantification of the relative proportions of CD11b^+^ Gr1^hi^ and CD11b^+^ Gr1^low^ populations in independent preparations. (C) Expression of CD11c and MHC II on total CD11b^+^ cells or in Gr1^hi^ and Gr1^low^ populations. Gray areas represent fluorescence minus one (FMO) controls. Data show representative results from at least four independent experiments.

**Figure 2:**
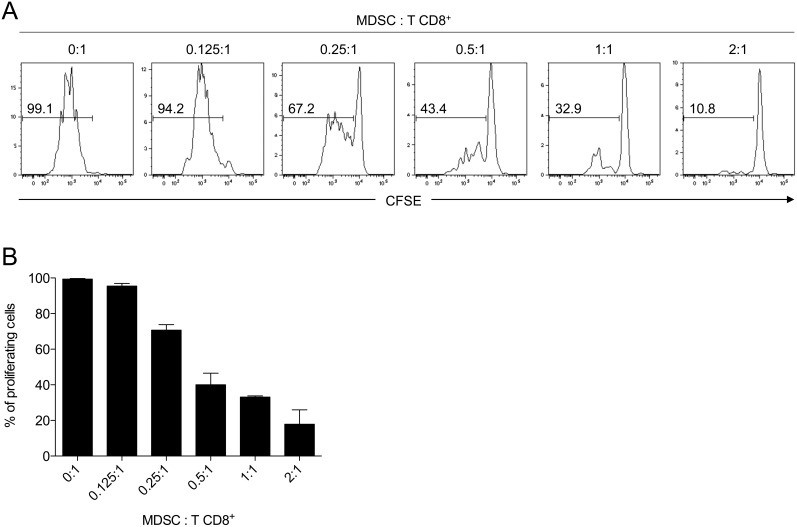
BM-derived MDSC efficiently inhibit T cell proliferation *in vitro.* CD8^+^ T cells were purified from OT-1 transgenic mice and labeled with CFSE before anti-CD3/CD28 bead stimulation. MDSC generated in vitro with GM-CSF and IL-6 were added to T cells at different ratios. After three days of culture, the percentage of proliferating cells (CFSE^low^) in CD8^+^ cells was assessed by flow cytometry. Representative histograms of CFSE dilution (A) and quantification of triplicates for each condition are shown (B). Data are representative of three independent experiments.

Based on the method described by Marigo et al. [32], we cultured BM cells from naive mice with GM-CSF and IL-6 and examined their phenotype after 4 days. We routinely obtained >90% of CD11b^+^ cells that could be subdivided in Gr1^hi^ and Gr1^low^ cells (Figure 1A and B). Gr1^low^ cells, which contain the majority of CD11c^+^ cells (Figure 1C), were shown to exhibit the highest suppressive activity [32]. Attributing the term MDSC to immature myeloid cells requires the demonstration of an immunosuppressive function, at least in vitro. As shown in Figure 2A and B, BM cells cultured with GM-CSF and IL-6 efficiently prevented CD8^+^ T cell proliferation in a dose-dependent manner, reaching >80% inhibition at a ratio of 2∶1 (MDSC:T cells).

**Figure 3:**
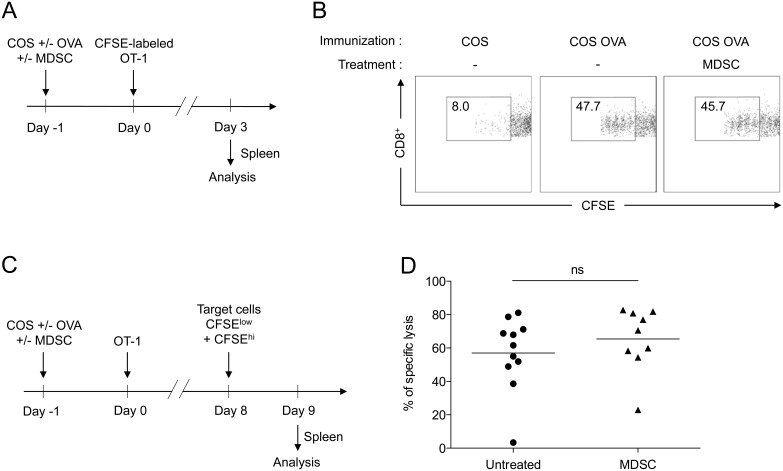
Adoptive transfer of BM-derived MDSC does not alter antigen-specific CD8^+^ T cell responses. (A–B) In vivo proliferation assay: COS cells transfected with a plasmid coding for GFP fused to the OVA_257–264_ peptide (COS OVA) or GFP alone (COS) were injected i.v. into mice with or without MDSC generated in vitro with GM-CSF and IL-6. Responder CD8^+^ T cells purified from OT-1 TCR-transgenic mice were labeled with CFSE and injected i.v. the following day. After 3 days, spleens of recipient mice were harvested to assess CFSE dilution by flow cytometry. Data are representative of two experiments. (C–D) In vivo cytotoxicity assay: CD8^+^ T cells purified from OT-1 TCR-transgenic mice (non labeled with CFSE) were injected in COS GFP/OVA-immunized mice as described above. After 8 days, CFSE-labeled CD45.1^+^ target cells either loaded with OVA_257–264_ (CFSE^hi^) or control (CFSE^low^) peptides were injected. Specific lysis was determined the next day by flow cytometry by measuring the relative proportion of each population in the spleen of MDSC-treated or untreated mice compared to non-immunized mice. Data show results from four independent experiments with 9 to 11 mice per group.

We then examined the suppressive potential of these MDSC in vivo. In order to best reproduce a T cell response triggered by a cellular antigen, we immunized mice with COS cells transfected with a plasmid encoding a non-secreted fusion protein linking the ovalbumin peptide SIINFEKL (OVA_257–264_) to GFP. In this system, the OVA peptide is presented to CD8^+^ T cells by recipient APCs on their MHC class I molecules through the processes of phagocytosis and antigen cross-presentation. The injection of CD8^+^ T cells from TCR-transgenic OT-1 mice then allows the monitoring of an antigen-specific T cell reponse in vivo, as depicted in Figure 3A and C. Immunization with OVA-expressing COS cells resulted in a strong CD8^+^ T cell proliferation while control COS cells did not. Concomitant adoptive transfer of MDSC and immunization did not prevent this proliferation (Figure 3B). We then hypothesized that, rather than significantly altering proliferation, MDSC could influence their differentiation into CTLs. However, as shown in Figure 3D, MDSC failed to impact antigen-specific T cell cytoxicity.

**Figure 4:**
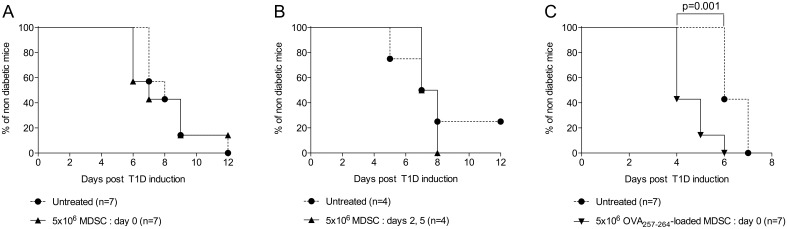
Adoptive transfer of BM-derived MDSC does not prevent the development of autoimmune diabetes. Type 1 diabetes was induced in RIP-mOVA mice by injecting (i.v.) naive CD8^+^ T cells from OT-1 TCR-transgenic mice together with an anti-OVA polyclonal antibody (i.p.). Blood glycemia was monitored every day during at least 12 days. Indicated numbers of MDSC generated in vitro with GM-CSF and IL-6 were adoptively transferred at day 0 (A) or at days 2 and 5 (B). Alternatively, MDSC were loaded with the OVA_257–264_ peptide before injection at day 0 (C). In each experiment, MDSC-treated mice were compared to a group of untreated mice.

To assess the effect of MDSC adoptive transfer in a more physiological context, we made use of a model of type 1 diabetes [34], [35] in which autoimmunity is induced by the injection of CD8^+^ OT-1 T cells in conjunction with a polyclonal anti-OVA antibody (Ab) into RIP-mOVA transgenic mice (membrane OVA is expressed as a neo-antigen by the pancreatic beta cells under the rat insulin promoter). In our hands, and as previously established [34], virtually all mice become diabetic within 5 to 12 days. Single adoptive transfer of MDSC on the day of OT-1 and Ab injection did not prevent diabetes development (Figure 4A). We reasoned that MDSC might benefit from an inflammatory milieu to stably exert their suppressive function on T cells. However, neither two consecutive adoptive transfers of MDSC, 2 and 5 days after OT-1 and Ab injection (Figure 4B), nor a single injection at day 5 using twice as much cells (data not shown) significantly impinged on the progression of the disease. Finally, we tested whether the loading of MDSC with the antigenic peptide before injection could potentiate the suppression by promoting their interaction with the diabetogenic T cells. Strikingly, this approach seemed to rather exacerbate the development of the disease, since treated mice developed accelerated diabetes compared to control mice (Figure 4C).

**Figure 5:**
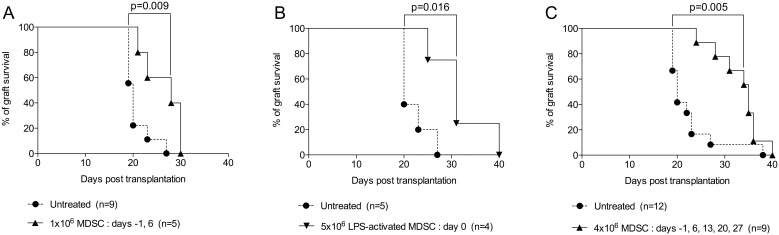
Adoptive transfer of BM-derived MDSC prolongs skin allograft survival. (A) Male skin grafts were transplanted onto females recipients treated or not at days −1 and 6 post-transplantation, with one million autologous (female) MDSC generated in vitro with GM-CSF and IL-6. (B) Alternatively, LPS was added in vitro for the last 5 hours of the MDSC culture and five million cells were injected at day 0. (C) Male skin grafts were transplanted onto females recipients treated or not at days −1, 6, 13, 20 and 27 post-transplantation, with four million autologous (female) MDSC generated in vitro with GM-CSF and IL-6. Graft survival was monitored every other day from day 7 post-transplantation.

Next, we tested whether adoptive transfer of MDSC could modulate a polyclonal response in the context of allograft rejection. As shown in Figure 5A, while male skin grafts transplanted onto female recipients were rejected within 19 to 28 days, two injections of syngenic (female) MDSC, the day before transplantation and at day 6 post-transplantation, were sufficient to prolong graft survival. A single injection of LPS-activated MDSC (LPS was added to the MDSC culture for the last 5 hours) on the day of transplantation similarly achieved a significant outcome (Figure 5B). However, this effect was markedly and reproducibly enhanced with five weekly consecutive injections, leading to graft survival up to 40 days (Figure 5C).

**Figure 6:**
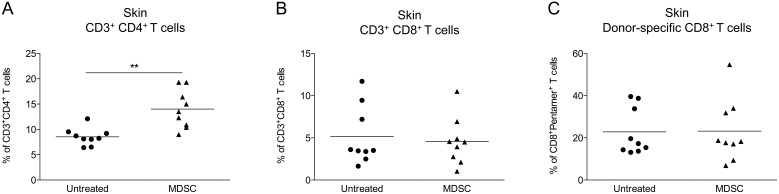
Adoptive transfer of BM-derived MDSC does not prevent lymphocyte infiltration in skin allografts. Male skin grafts were transplanted onto females recipients treated or not at days −1, 6 and 13 post-transplantation with four million autologous (female) MDSC generated in vitro with GM-CSF and IL-6. Skin grafts were harvested 14 days after transplantation and infiltrated leukocytes were analyzed by flow cytometry. Results are expressed in percentages of CD3^+^ CD4^+^ T cells (A), CD3^+^ CD8^+^ T cells (B) and donor-specific Pentamer^+^ cells among CD8^+^ T cells (C). Data show results from two independent experiments with 4 to 9 mice per group. **p<0.01.

**Figure 7:**
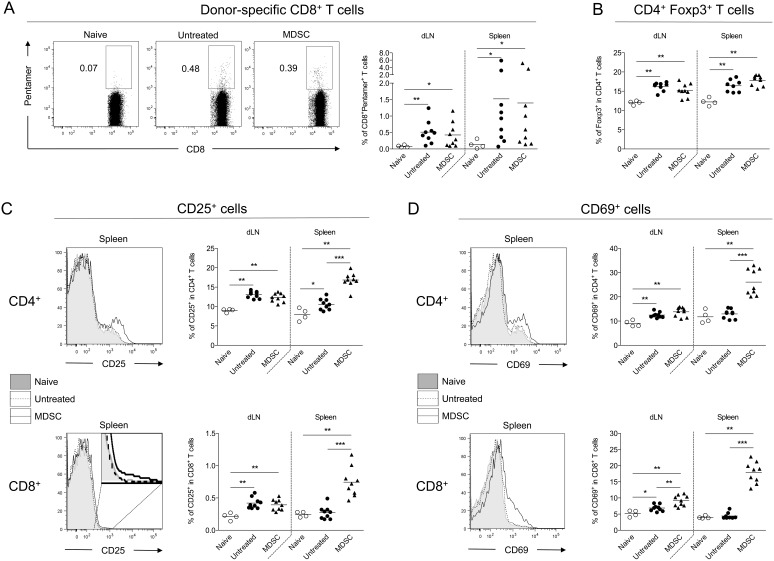
Adoptive transfer of BM-derived MDSC is associated with increased numbers of CD25^+^ and CD69^+^ cells, mainly in the spleen. Male skin grafts were transplanted onto females recipients treated or not at days −1, 6 and 13 post-transplantation with four million autologous (female) MDSC generated in vitro with GM-CSF and IL-6. Draining lymph nodes and spleen were harvested from skin-grafted mice 14 days after transplantation or from naive mice for flow cytometry analysis. (A) Representative staining and quantification of donor-specific Pentamer^+^ CD8^+^ T cells in naive or skin-grafted mice. (B) Quantification of FoxP3^+^ cells among CD3^+^ CD4^+^ T cells. (C, D) Representative stainings and quantifications of CD25^+^ (C) and CD69^+^ (D) among CD3^+^ CD4^+^ or CD3^+^ CD8^+^ T cells. Data show results from two independent experiments with 4 to 9 mice per group. *p<0.05, **p<0.01, ***p<0.001.

**Figure 8:**
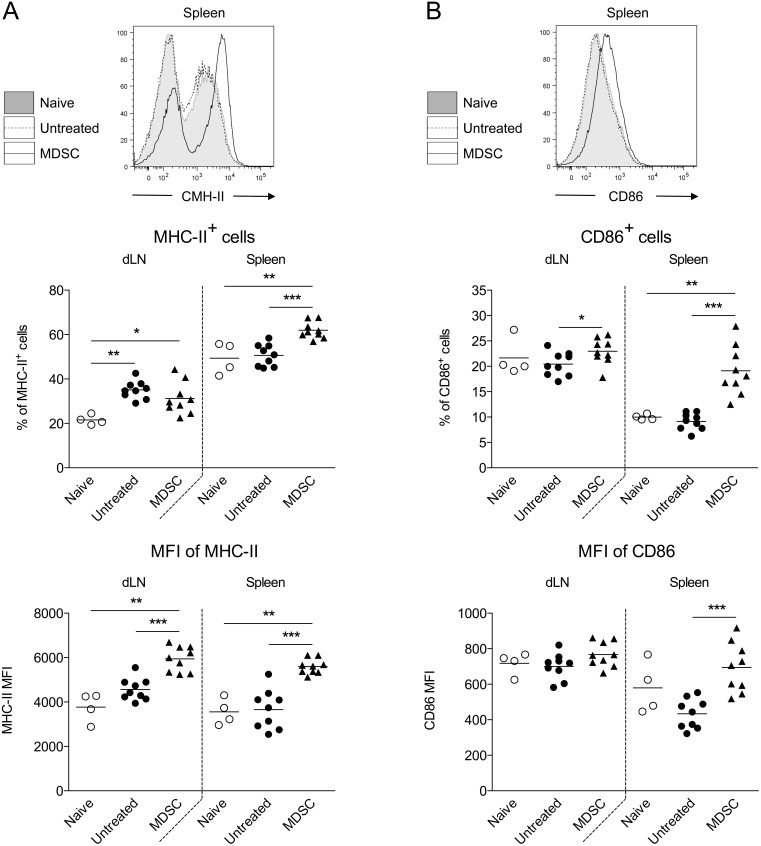
Adoptive transfer of BM-derived MDSC is associated with increased numbers of MHC II^+^ and CD86^+^ cells, mainly in the spleen. Male skin grafts were transplanted onto females recipients treated or not at days −1, 6 and 13 post-transplantation with four million autologous (female) MDSC generated in vitro with GM-CSF and IL-6. Draining lymph nodes and spleen were harvested from skin-grafted mice 14 days after transplantation or from naive mice for flow cytometry analysis. Representative stainings and quantifications of MHC II^+^ (A) and CD86^+^ (B) cells in naive or skin-grafted mice. Data show results from two independent experiments with 4 to 9 mice per group. *p<0.05, **p<0.01, ***p<0.001.

To understand the beneficial effect of MDSC adoptive transfers on skin graft survival, we investigated the immune cell composition directly in the graft as well as in the draining lymph nodes and in the spleen, two weeks post-transplantation (after three weekly injections of MDSC). Few or no injected MDSC were detected (using the congenic marker Ly5.1) suggesting that these cells are rapidly eliminated or preferentially home to a distinct location than the skin graft, the draining lymph nodes or the spleen. Surprisingly, we found that skin grafts from both untreated and MDSC-treated mice showed similar numbers of total infiltrated leucocytes (data not shown). In fact, the proportion of CD4^+^ T cells was even increased in MDSC-treated mice (Figure 6A) whereas no difference was observed for CD8^+^ T cells (Figure 6B). In addition, donor-specific CD8^+^ T cells were found in similar numbers both in skin grafts (Figure 6C) and in the periphery (Figure 7A). The proportions of CD19^+^ B cells, CD3^−^ NK1.1^+^ NK cells, CD3^+^ CD4^+^ or CD3^+^ CD8^+^ T cells were not altered by MDSC adoptive transfers (data not shown). FoxP3^+^ cell numbers among CD4^+^ T cells were increased in skin-grafted mice compared to naive mice but no significant differences were observed between untreated and MDSC-treated mice (Figure 7B). As expected, increased numbers of CD25^+^ and CD69^+^ T cells were detected mostly in the draining lymph nodes of skin graft recipients compared to naive mice (Figure 7C and D). However, MDSC adoptive transfers did not prevent this activation phenotype. On the contrary, we observed a dramatic increase of CD25^+^ and CD69^+^ T cell numbers in MDSC-treated mice, almost exclusively in the spleen (Figure 7C and D), a phenomenon that was also associated with increased numbers of MHC II^+^ and CD86^+^ cells (Figure 8A and B). Thus, MDSC adoptive transfers, rather than specifically suppressing the allogenic immune response, appears to induce a state of systemic activation that correlates with prolongation of skin graft survival.

## Discussion

Compelling evidence from animal models suggest a great potential of MDSC adoptive transfer for preventing graft rejection or treating autoimmune disorders. For example, MDSC from tumor-bearing mice have been shown to prevent the onset of type 1 diabetes when co-transferred with diabetogenic CD4^+^ T cells [7]. Similarly, MDSC purified from LPS-treated mice are capable of prolonging skin allograft survival [18]. These findings prompted us to embark on a study to assess their therapeutic potential in mouse models of autoimmunity and transplant rejection. A translational view implicates the development of a clinically acceptable method for the production of these cells. Marigo et al. provided convincing data highlighting the high suppressive activity of MDSC generated from BM cells using GM-CSF supplemented with IL-6, for the in vivo inhibition of T cell responses as well as the prevention of allogenic islet rejection [32].

While we succeeded in producing in vitro suppressive CD11b^+^ cells using this approach, we did not observe any alteration of in vivo antigen-specific CD8^+^ T cell responses or autoimmune diabetes development after adoptive transfer of these cells. It is important to note that this stringent in vivo experimental procedure involves a high number of strongly reactive monoclonal (OT-1 TCR transgenic) T cells. Furthermore, the expansion of diabetogenic CD8^+^ T cells is not the result of homeostatic proliferation since RIP-mOVA mice are non-irradiated lymphosufficient hosts, but strictly depends on robust Fc receptor-mediated OVA cross-presentation by DCs [34].

Surprisingly, we found that loading MDSC with the neo-antigen OVA peptide rather exacerbated than dampened the development of the disease. This observation was reproduced using in vivo cytotoxicity assay (data not shown). The culture of whole BM cells with GM-CSF and IL-6 results in an heterogeneous mixture of myeloid cells, a fraction of them likely bearing the potential to differentiate into highly immunogenic DCs. Additional factors, such as PGE_2_ [36] or subset separation before injection, may help to maintain a suppressive homogeneity. The use of few markers expressed at the surface of MDSC obviously does not satisfy the requirement of a pure and stable suppressive population. In this regard, CD11b^+^ Gr1^+^ cells have also been described as immunostimulatory during tumor growth [37] or autoimmunity [38]. Taken together, these observations could raise doubts over the safety of BM-derived myeloid cell transfer, potentially detrimental in specific inflammatory situations.

The transplantation of male skin onto female recipients mounts a progressive expansion of low frequency polyclonal T cell clones leading to graft rejection. In this model, in contrast to type 1 diabetes, we found that multiple injections of MDSC significantly prolonged graft survival. It is tempting to speculate that a continuous treatment could result in long-term acceptance of the graft, as shown by Marigo et al. in pancreatic islet transplantation [32]. Of note, two injections of MDSC were not sufficient to prevent or delay rejection of complete mismatch skin grafts (Balb/c onto C57BL/6 mice, data not shown) pointing to the limit of these in vitro generated MDSC to impinge, by themselves, on a strong allogenic response, yet in the same manner as in vitro expanded Tregs, alone, failed to provide significant graft prolongation in a complete mismatch setting, in lymphosufficient mice [39].

These results also emphasize the need for identifying strategies to increase and preserve the suppressive ability of MDSC after transfer in order to reduce the frequency of injections. Indeed, MDSC have been shown to rapidly differentiate into mature myeloid cells in the absence of tumor-derived factors or sustained inflammation [40], [41]. In this regard, Greifenberg et al. originally demonstrated that LPS + IFN-γ combination considerably augmented the suppressive capacity of MDSC by impairing DC differentiation [42]. Similarly, Highfill et al. showed that addition of IL-13 in BM cells cultured with GM-CSF and G-CSF resulted in the production of potently suppressive MDSC that efficiently inhibited graft-versus-host disease [43]. Thus, amongst other strategies that have been reported to promote MDSC activation/expansion [5], our results support the relevance of this approach since a single injection of LPS-activated MDSC was sufficient to induce a significant prolongation of graft survival. It remains to be evaluated whether additional injections of these activated cells will reinforce this beneficial effect and if the addition of IFN-γ (or other cytokines) could further boost their suppressive function in vivo.

Mechanistically, we have found that the beneficial effect of MDSC infusions on skin graft survival was paradoxically not explained by a decrease of donor-specific T cell response but rather associated with an over-activation of T cells and antigen presenting cells. The fact that this observation was prominently made in the spleen suggests that MDSC transfers could create a window of systemic exhaustion in the immune system allowing the allogenic graft to survive, a phenomenon that would terminate immediately after cessation of the therapy, then excluding any mechanism of long term tolerance. Thus, while this effect is associated with delayed graft rejection in the setting of transplantation, it appears inefficient or rather detrimental during the developpement of a fast and potent autoimmune response. These differential outcomes stress the need to carefully evaluate MDSC adoptive transfer therapies, or any other approaches, by using carefully chosen models in relation with the clinical aim.

Interestingly, Treg therapy alone in lymphosufficient hosts, even in an antigen-specific fashion, similarly fails to induce a long-term protection from allograft rejection [39], [44]. The combination of MDSC and Treg cell therapies could result in a synergistic effect. Indeed, numerous reports have shown that MDSC promote the development and homeostasis of Tregs over CD4^+^ T effector cells [45], notably in the context of type 1 diabetes [7], [8]. Moreover, MDSC can capture and present exogenous antigens to their MHC class II molecules which can be drastically upregulated upon IFN-γ stimulation [46]. Treg accumulation has also been attributed to monocytic suppressive cells [19]. Thus, in spite of a recent study that challenged this view concerning granulocytic MDSC [47], these results generally argue for a beneficial interplay between Tregs and MDSC that could be relevant in the context of cellular therapy. Athough similar levels of FoxP3^+^ Treg were found in the draining lymph nodes or spleen of MDSC-treated mice, the detection of a potential beneficial effect of MDSC on these cells in the periphery will probably require the examination of the (donor) antigenic specificity. Moreover, it will be interesting to determine whether the increase of CD4^+^ T cells that we observed in the skin grafts of MDSC-treated mice could reflect an influx of Treg that would be mostly specific for the donor antigens.

In summary, in the present study, we have compared the potential of in vitro generated MDSC adoptive transfer in relevant and distinct in vivo models of immune response. Our data highlight the need to refine the in vitro generation of homogeneous, stable and strongly suppressive myeloid cells before considering a therapeutic approach, most likely with combination treatments.

## Materials and Methods

### Ethics Statement

This study was carried out in strict accordance with the protocol approved by the Commitee on the Ethics of Animal Experiments of Pays de la Loire (Ref: CEEA.2012.211 and CEEA.2013.9).

### Mice

C57BL/6 mice were purshased from Janvier (France). RIP-mOVA (C57BL/6-Tg(Ins2-TFRC/OVA)296Wehi/WehiJ) transgenic mice [48] were purchased from The Jackson Laboratory (Bar Harbor, ME, USA). For this line, hemizigous mice were maintained in the laboratory by breeding transgenic mice, selected by PCR genotyping, with wild-type C57BL/6 mice. OT-1 TCR-transgenic mice (C57BL/6-Tg(TcraTcrb)1100Mjb/Crl) [49] and Ly5.1 congenic mice (B6.SJL-*Ptprc^a^ Pepc^b^*/BoyCrl) were purshased from Charles Rivers (France).

### Reagents

Murine GM-CSF was from Peprotech (Neuilly-sur-Seine, France). IL-6 and LPS were from Sigma-Aldrich (Saint-Quentin Fallavier, France). CFDA-SE (CFSE) was from Molecular Probes (Montluçon, France). OVA (SIINFEKL) and Smcy (KCSRNRQYL) peptides were from PolyPeptide (Strasbourg, France). Anti mouse CD11b biotin (M1/70) (used with streptavidin APC or streptavidin APC-Cy7), CD11b APC-Cy7 (M1/70), CD11c PE-Cy7 (HL3), I-A^b^ FITC (AF6-120.1), Gr1 PE (Ly6C/G, RB6-8C5), CD45.1 APC (A20), CD45.2 APC-Cy7 (104), CD45.2 PerCP-Cy5.5 (104), CD19 APC (1D3), NK1.1 PE (PK136), CD3ε PerCP-Cy5.5 (145-2C11), CD3ε Pacific Blue (500A2), CD3ε FITC (145-2C11), CD4 PE-Cy7 (RM4-5), CD8α Pacific blue (53-6.7), CD8α APC-Cy7 (53-6.7), CD8α PerCP-Cy5.5 (53-6.7), FoxP3 Alexa Fluor647 (MF23), CD25 PE (704), CD69 FITC (H1.2F3), and CD86 FITC (B7.2, GL1) were from BD PharMingen (Le Pont de Claix, France). Male antigen UTY-specific CD8^+^ T cells were detected using a PE labelled Pro5 MHC Pentamer (H-2D^b^, WMHHNMDLI) (ProImmune Limited, Oxford, UK).

### Generation of BM-derived MDSC

MDSC were generated as previously described [32]. Tibias and femurs from C57BL/6 mice were removed and BM was flushed. Red blood cells (RBCs) were lysed with ammonium chloride. To obtain BM-derived MDSC, 2.5×10^6^ cells were plated into dishes with 100 mm diameter in 10 mL of complete medium, which consisted of 10% heat-inactivated fetal bovine serum (Lonza, Levallois, France), nonessential amino acids, 1 mM sodium pyruvate, 10 mM HEPES (all from Gibco, Saint Aubin, France), 2 mM glutamine and 50 µM β-mercaptoethanol (both from Sigma-Aldrich) in DMEM base (Gibco). Medium was supplemented with GM-CSF (40 ng/ml) and IL-6 (40 ng/ml) cytokines. Cells were maintained at 37°C in 5% CO_2_-humidified atmosphere. After 4 days, cells were washed twice before flow cytometry analysis, in vitro culture or in vivo injection. In some experiments, LPS was added (1 µg/mL final) for the last 5 hours of the culture.

### *In vitro* Proliferation Assay

Responder CD8^+^ T cells were purified (CD8a^+^ T cell Isolation Kit II, Miltenyi Biotec, Paris, France) from spleens of naive C57BL/6 mice, labeled with CFSE and plated at the concentration of 2×10^4^ cells/mL in 96-well round bottom plate in 200 µL final of complete medium. Anti-CD3/28 microbeads (Life Technologies, Saint Aubin, France) were used at a 1∶1 ratio and increased numbers of BM-derived MDSC were added. After 3 days, CFSE dilution in CD8^+^ T cells was analyzed by flow cytometry.

### Immunization with OVA-expressing COS Cells

COS cells were transfected (Lipofectamine Transfection Reagent, Life Technologies) with plasmids (pCI-neo backbone, Promega, Charbonnières-les-Bains, France) coding for GFP alone or GFP fused to OVA_257–264_ sequence (SIINFEKL peptide) at N-terminal. After 48 hours, COS cells expressing GFP alone (control COS) or OVA_257–264_-GFP fusion protein (COS OVA) were trypsinized and washed in PBS before i.v. injection (1×10^5^ cells). Transfection efficiency routinely reached 40–50% of GFP^+^ cells.

### *In vivo* Proliferation Assay

Experimental scheme is depicted in Figure 3A. C57BL/6 mice were immunized with control COS or COS OVA cells and co-injected (i.v.) or not with 6.5×10^6^ BM-derived MDSC. The next day, 5×10^6^ CD8^+^ T cells purified (CD8a^+^ T cell Isolation Kit II, Miltenyi Biotec) from pooled spleens and lymph nodes of OT-1 TCR-transgenic mice were labeled with CFSE and injected (i.v.). After 3 days, spleens were harvested and CFSE dilution in injected CD8^+^ T cells was analyzed by flow cytometry.

### *In vivo* Cytotoxicity Assay

Experimental scheme is depicted in Figure 3C. C57BL/6 mice were immunized with control COS or COS OVA cells and co-injected (i.v.) or not with 5×10^6^ BM-derived MDSC. The next day, 0.25×10^6^ CD8^+^ T cells purified (CD8a^+^ T cell Isolation Kit II, Miltenyi Biotec) from pooled spleens and lymph nodes of OT-1 TCR-transgenic mice were injected (i.v.). After 8 days, spleens cells from Ly5.1 mice (CD45.1^+^ cells) were labeled with 4 µM or 0.2 µM of CFSE to obtain CFSE^hi^ and CFSE^lo^ populations respectively loaded with control Smcy and OVA_257–264_ peptides and were injected (i.v.) at a 1∶1 ratio (1.6×10^6^ cells for each population). The next day, spleens were harvested to measure the relative proportions of each population within CD45.1^+^ cells by flow cytometry. Specific lysis was determined by calculating the percentage of decrease of the CFSE^hi^ population in immunized mice compared to non-immunized mice.

### Induction of Autoimmune Diabetes

Diabetes was induced in RIP-mOVA mice as previously described [34]. Briefly, 6 to 8 week-old RIP-mOVA mice were injected intravenously with 5×10^6^ CD8^+^ T cells purified (CD8a^+^ T cell Isolation Kit II, Miltenyi Biotec) from pooled spleens and lymph nodes of OT-1 TCR-transgenic mice together with intraperitoneal administration of 1 mg anti-OVA IgG. Anti-OVA serum was obtained from ovalbumin (OVA)-hyperimmunized rabbits (Covalab, Villeurbanne, France) and IgG were purified by protein A affinity chromatography. Endotoxin-free OVA protein was from Profos (Regensberg, Germany). Blood glucose levels were measured with a StatStrip Xpress Glucose/Ketone Meter monitoring system (Nova Biomedical, Les Ulis, France). Mice were considered diabetic after two consecutive measurements >250 mg/dL.

### Skin Transplantation

Mice were anesthetized with a mixture of 5% xylazine (Rompun) and 18% ketamine in PBS (170 µL) injected intraperitoneally (8.5 mg/kg of xylazine and 76.5 mg/kg of ketamine per mouse). Square skin grafts (1 cm^2^) were prepared from the tail of male wild-type C57BL/6 donors and transplanted on the shaved left flank of C57BL/6 female recipients. The grafts were fixed to the graft bed with 10–12 interrupted sutures and were covered with protective tape. The first inspection was carried out seven days later and graft survival was monitored every other day. Rejection was defined as complete sloughing or a dry scab.

### Analysis of Cellular Populations in Skin Graft Recipients

Fourteen days after skin transplant, mice were sacrificed and draining lymph nodes, spleen and skin graft were harvested. Skin grafts were processed using collagenase D (Sigma-Aldrich) for 45 min at 37°C. Cells were fluorescently labeled and cellular populations were analyzed by flow cytometry.

### Statistical Analysis

Statistical analyses were performed with Graphpad Prism 5.0 (La Jolla, CA, USA) using the Mann-Whitney test. Survival rates were compared using the Log-rank (Mantel-cox) test. Statistical significance was defined as p<0.05.
